# Cardiovascular Risk in Autoimmune Diseases: Mechanisms, Management, and Emerging Evidence

**DOI:** 10.7759/cureus.91897

**Published:** 2025-09-09

**Authors:** Maheen Bhangwar Baloch, Chris Alphonse, Nehal Baldev, Genesis Marie Nin-Arroyo, Rohith Keezhath, Anahita Behara, Swati Khurana, Stephanie Nwokeji, Bashir Imam, Yozahandy A Abarca-Pineda

**Affiliations:** 1 Medicine and Surgery, Ziauddin University, Karachi, PAK; 2 Acute Medicine, Jubilee Mission Medical College and Research Institute, Thrissur, IND; 3 General Medicine, Gujarat Medical Education and Research Society (GMERS) Medical College and Hospital, Junagadh, IND; 4 Medicine, Universidad Autónoma de Guadalajara, Guadalajara, MEX; 5 General Internal Medicine, Medway Maritime Hospital, Gillingham, GBR; 6 Medicine, Kamineni Institute of Medical Sciences, Hyderabad, IND; 7 Pathology, MVJ Medical College and Research Hospital, Bengaluru, IND; 8 Oncology, Rush University Medical Center (RUSH) MD Anderson Cancer Center, Chicago, USA; 9 Internal Medicine, Jackson Park Hospital and Medical Center, Chicago, USA; 10 Internal Medicine, Hospital Médica Sur, Mexico City, MEX

**Keywords:** atherosclerosis, autoimmune diseases, cardiovascular risk, chronic inflammation, endothelial dysfunction, il-17 inhibitors, lifestyle intervention, methotrexate, risk stratification, tnf-α inhibitors

## Abstract

Cardiovascular disease is a leading cause of morbidity and mortality among patients with autoimmune disorders. Chronic inflammatory conditions such as rheumatoid arthritis, systemic lupus erythematosus, psoriasis, and inflammatory bowel disease are associated with accelerated atherosclerosis and other vascular complications. Persistent inflammation, dysregulated cytokine networks, and autoantibody-mediated pathways contribute to endothelial dysfunction, pro-thrombotic states, and metabolic disturbances. In addition to traditional risk factors, disease-specific elements such as chronic glucocorticoid exposure and altered lipid profiles influence overall risk. Management strategies emphasize the control of systemic inflammation alongside standard cardiovascular prevention measures. Biologic therapies targeting tumor necrosis factor, interleukin-6, and other inflammatory mediators may attenuate cardiovascular risk by slowing plaque progression. Lifestyle interventions and pharmacologic measures (e.g., statins and antihypertensives) are crucial to mitigate coexisting risks. Emerging evidence from recent cohort studies and clinical trials suggests that these targeted treatments can improve cardiovascular outcomes in autoimmune populations. Advanced vascular imaging and novel biomarkers now allow the earlier detection of subclinical atherosclerosis. Ongoing research into genetic predisposition, the gut microbiome, and new immune pathways offers deeper insight into risk mechanisms. By integrating current pathophysiologic understanding with evolving clinical data, these insights inform strategies for optimizing long-term cardiovascular health in patients with autoimmune disease.

## Introduction and background

Autoimmune disease (AD) happens when our immune response creates antibodies to self-antigens, failing to distinguish between foreign and self. Because they can affect nearly every organ and tissue, these conditions are regarded as systemic diseases [1]. Chronic inflammation is one of the most predominant characteristics, leading to progressive damage and increasing the risk of complications, such as cardiovascular diseases (CVD) [2-4].

With the increasing amount of research available, more data arise suggesting a link between AD and cardiovascular risks. Patients suffering from an AD present with a significantly higher risk of developing cardiovascular events, often at younger ages than their counterparts [5]. This is particularly true for women who, based on the hormonal cycle, present a predisposition for CVD when having one or more ADs [6,7]. Patients with AD must get cardiovascular screening and intervention early.

Therefore, ADs are increasingly considered an independent risk factor for cardiovascular events, particularly in women [7]. This growing correlation has implications for public health and highlights the need for earlier cardiovascular screening and intervention in these populations.

The pathophysiological relationship between AD and CVD is complex, yet it is becoming increasingly well understood. Sharing common inflammatory mechanisms, including activating Th1 and Th17 cells, promotes a cascade involving tumor necrosis factor (TNF), interleukin (IL)-12, and IL-23 cytokines [8,9]. These molecules contribute to endothelial dysfunction, genetic factors, insulin resistance, oxidative stress, and adipokine secretion, which are the etiopathogenic factors for CVD [10,11].

TNF-α has been identified as a pivotal cytokine in the pathogenesis of atherosclerosis and ADs [12], such as psoriasis and systemic lupus erythematosus (SLE). Supporting biologic therapies targeting TNF-α has been shown to reduce cardiovascular risk in patients suffering from chronic inflammatory diseases [10].

Additionally, conventional immunosuppressants such as methotrexate, often used as the first-line treatment for psoriasis and rheumatoid arthritis, have demonstrated protective CVD effects. Consider the case of patients treated with 20 mg per week of methotrexate, who showed decreased carotid intima-media thickness compared with lower doses [10]. 

However, newer biologics, such as IL-12/23 inhibitors, have raised concern. The biologic briakinumab was withdrawn due to an observed increased incidence of major adverse cardiovascular events (MACE), though the evidence remains inconclusive and requires further investigation [9,12]. 

This review explores the cardiovascular risks associated with various autoimmune conditions and discusses the most recent evidence concerning their pathophysiological overlap and management strategies. It underscores the importance of incorporating routine cardiovascular risk assessment into the standard care of patients with AD.

## Review

Epidemiological association between ADs and cardiovascular risk

ADs are known for their complex and unpredictable effects on the body. These conditions occur when the immune system starts attacking our tissues instead. While often overshadowed by illnesses like cancer or diabetes, ADs can quietly cause damage to one of the body's most vital systems: the cardiovascular system.

People dealing with ADs face a significantly higher risk of heart issues. The root problem often lies in long-standing inflammation. Imagine a low-grade fire that keeps burning inside the body: it gradually damages blood vessels and overworks the heart. Over time, this silent inflammation can lead to strokes, heart attacks, arrhythmias, and even heart failure [13,14].

This risk is even greater when a person has more than one AD. Yet, many patients aren't routinely assessed for cardiovascular risk, partly because traditional screening tools don't take inflammation into account [15].

Psoriasis is often perceived as a purely skin disorder, but it is in fact a systemic inflammatory disease affecting multiple organ systems. In fact, people with psoriasis have nearly a 50% higher chance of having a heart attack than those without it [16,17]. That is why early cardiovascular evaluation and changes in lifestyle, such as regular exercise, balanced nutrition, and quitting smoking, are essential. These measures don't just help the heart; they improve overall disease control too [18].

Globally, psoriasis affects approximately 2-5% of the population, with 20-30% of affected individuals developing psoriatic arthritis, highlighting its systemic nature [12]. Mounting epidemiological evidence indicates that psoriasis serves as an independent risk factor for various cardiometabolic diseases, including myocardial infarction, stroke, hypertension, diabetes mellitus, and metabolic syndrome [10,19]. The risk of cardiovascular morbidity and mortality is notably higher in patients with severe or early-onset disease.

Data indicate that individuals under the age of 50 with moderate-to-severe psoriasis exhibit nearly twice the risk of developing arteriosclerotic disease compared to the general population, even after adjusting for traditional risk factors [12]. Males are more predisposed to ischemic heart disease, whereas females with psoriasis show a higher incidence of ischemic stroke and metabolic complications. Among elderly populations, although vascular risk does not differ significantly from age-matched controls, the burden of comorbid risk factors remains elevated [12].

The connection between dermatological and cardiovascular pathology is underpinned by shared inflammatory pathways, particularly involving Th1 and Th17 cytokines such as TNF-α, IL-6, IL-17, and IL-23 [10]. These inflammatory mediators contribute to endothelial dysfunction, vascular inflammation, and atherogenesis. Additionally, systemic features such as oxidative stress, angiogenesis, insulin resistance, and hypercoagulability are commonly observed in both psoriasis and CVD [10,19].

Lifestyle and behavioral risk factors further compound cardiovascular risk in psoriasis patients. Smoking, excessive alcohol intake, and central obesity are significantly more prevalent in this population. Dyslipidemia, particularly elevated low-density lipoprotein (LDL), reduced high-density lipoprotein (HDL), and increased triglycerides, is observed in both adults and children with psoriasis, exacerbating cardiovascular vulnerability [12].

Biologic therapies targeting inflammatory cytokines have demonstrated promising effects not only on skin manifestations but also on cardiovascular outcomes. Treatments directed at TNF-α, IL-17, and IL-12/23 have been associated with reductions in systemic inflammation and, in some studies, lower rates of MACE. However, certain biologics, such as IL-12/23 inhibitors, may require caution in patients with pre-existing cardiac conditions due to early treatment-associated cardiovascular event risks [10,12].

Pathophysiological mechanisms linking AD and CVD

ADs such as SLE, psoriasis, rheumatoid arthritis, and systemic sclerosis have shown a significant risk of CVD. This risk cannot be explained by known cardiovascular risk factors, which implies that disease-specific mechanisms exist that accelerate atherogenesis and other cardiovascular complications [4,20].

Chronic systemic inflammation acts as a central driver of atherosclerosis in ADs. Pro-inflammatory cytokines, such as TNF-α and IL-6, lead to endothelial dysfunction and activation, primarily via the nuclear factor κ-B (NFκ-B) pathway. Activation of this pathway entails the enhanced expression of chemoattractants (such as C-C motif chemokine 2), adhesion molecules (such as vascular cell adhesion protein 1 (VCAM-1)), and pro-inflammatory cytokines, which all promote leukocyte infiltration, proliferation, and activation in the subendothelial space which supports the increased uptake of LDL by macrophages and causes the atherosclerotic plaques [21].

In rheumatoid arthritis, the elevation of cytokine IL-6 correlates with increased carotid intima-media thickness and coronary artery calcification [22]. Similarly, in SLE, when the systemic immune system activates, it produces a pro-inflammatory environment that increases the risk of early cardiovascular events, which is why young women with SLE have a 50-fold increased risk of myocardial infarction as compared to similar women without SLE [20,23].

Plaque morphology is altered by inflammation, which enhances rupture potential and thrombus formation. Macrophage-derived matrix metalloproteinases degrade fibrous caps of plaques, while inflammatory cytokines impair the function of endothelial progenitor cells, reducing vascular repair capacity [24].

A key early step in atherogenesis is endothelial dysfunction, which is significantly enhanced in autoimmune conditions. Inflammatory cytokines diminish nitric oxide bioavailability, which promotes vasoconstriction, leukocyte adhesion, and thrombosis [25]. In systemic sclerosis, vascular complications are compounded by obliterative vasculopathy and intimal proliferation, causing macro- and microvascular complications [4,26].

Direct immune-mediated vascular injury plays an important role. Autoantibodies like anti-endothelial cell antibodies, present in SLE and vasculitis, bind endothelial cells, triggering complement activation and cytotoxicity [21]. In antiphospholipid syndrome (APS), which is commonly associated with SLE, antiphospholipid antibodies increase thrombosis risk by platelet activation, tissue factor upregulation, and fibrinolysis [27].

Neutrophils and macrophages mediate oxidative stress, which is another contributing factor to endothelial injury. The endothelium is also damaged by reactive oxygen species (ROS). ROS also oxidize LDL and impair HDLs' anti-atherogenic functions, causing dysfunctional HDL [24,28].

Despite low or normal LDL levels during active inflammation in rheumatoid arthritis and SLE, the risk of CVD remains high. This can be explained by the changes in lipoproteins and inflammation-driven lipid deposition [23,29].

Immune complex deposition leads to complement activation and vascular inflammation in SLE, while early complement components cause immune complex clearance and chronic activation, which is shown by low C3 and C4, which increases vascular injury and is linked to a higher risk of cardiovascular events [20,25].

Each of the ADs contributes unique mechanisms. In rheumatoid arthritis, specific factors like epitope and anti-citrullinated protein antibodies are associated with endothelial dysfunction and coronary calcification [26,29]. In psoriasis, IL-17 and IL-23 promote vascular inflammation and atherosclerosis [24]. Systemic sclerosis causes macrovascular ischemia, which increases cardiac and pulmonary vascular morbidity [26].

In summary, all these disease-specific immunopathological pathways highlight the need for cardiovascular risk assessment and strategies to guide intervention in ADs. Recognizing and considering the importance of chronic inflammation and vascular pathology is essential for improving outcomes.

Clinical implications: screening and risk stratification

ADs are often associated with the development of cardiovascular manifestations, including atherosclerosis. Therefore, it is crucial to have an assessment of risk factors and manage those to prevent comorbidities. Screening could improve the outcome as CVD is a principal reason for fatality in these patients [30].

Various studies have shown that the risk of CVD in middle-aged female SLE patients was eight- or ninefold higher and was correlated with high blood pressure, age, and other SLE-linked factors [31]. In contrast to normal masses, the risk of CVE and morbidity is twofold higher in SLE patients without considering only the traditional risk factors for CVD [31]. Moreover, the tendency to develop pulmonary arterial hypertension (PAH) is well known in patients with ADs like rheumatoid arthritis, APS, mixed connective tissue disease (MCTD), SLE, progressive systemic sclerosis (PSS), dermatomyositis, and Sjögren's syndrome (SS) [31,32]. Thus, in comparison to the general population, the risk-bearing is high in patients with immune-mediated diseases driven by classical risk factors such as smoking, hypertension, dyslipidemia, obesity, cachexia, insulin resistance, and physical inactivity [33].

These factors are taken into consideration along with age and sex by many risk estimation models to estimate the 10-year risk of CVD, sparing acute and chronic inflammation markers as an independent predictor of cardiovascular risk. Although these grossly identify risk in general using the Systematic Coronary Risk Evaluation (SCORE) and Framingham Risk Score (FRS), the CVD risk is underestimated in the population of chronic immune diseases [34].

The use of CVD risk predictors like FRS or the American College of Cardiology (ACC) and American Heart Association (AHA) Pooled Cohort Risk Equation to be multiplied by 1.5 to account for the effect of rheumatoid arthritis on CVD risk is suggested by the European Alliance of Associations for Rheumatology (EULAR) guidelines [35]. Although there are established benefits of FRS, which include traditional risk factors for the general population, it does not explain the correlation of significant cardiac events in SLE patients with only those considerations [36]. 

To identify high-risk autoimmune patients for CVD, clinicians should focus on disease-specific factors, such as specific autoimmune conditions (e.g., systemic sclerosis, lupus, type 1 diabetes), elevated inflammatory markers, specific genetic predispositions, and the presence of subclinical atherosclerosis. Beyond traditional risk factors, incorporating novel assessments such as the clonal hematopoiesis of indeterminate potential (CHIP) testing, advanced imaging for arterial buildup, and monitoring for elevated heat-shock proteins (anti-HSP65) can help more accurately stratify risk [35].

Therapeutic interventions and cardiovascular impact

TNF-α Inhibitors

TNF-α inhibitors have been shown to confer notable cardiovascular benefits across the spectrum of immune-mediated inflammatory diseases. A meta-analysis found that TNF inhibitors were significantly associated with reduced risks of overall cardiovascular events, including myocardial infarctions, strokes, and MACE [12,37], though no significant effect on heart failure was observed [38].

Treatment with TNF-α inhibitors in psoriasis patients could reverse early atherosclerosis at the initial stage, presented as significantly reduced arterial intima-media thickness [10]. Different atherosclerosis‑associated biomarkers (E‑selectin, IL‑22, and hs-CRP) have been shown to drop significantly after a 12‑week therapy with adalimumab [38]. Retrospective cohort studies comparing topical and TNF therapies have shown that myocardial infarction and MACE are significantly lower in the latter cohort (HR-0.27) [39]. However, a cautious approach is required in the population with heart failure who generally have elevated TNF-α levels, which correlate with worse symptoms. In clinical trials involving patients with congestive heart failure, etanercept showed no benefit, whereas infliximab showed worsening heart failure. As a result, severe heart failure is a contraindication for TNF inhibitors in this subgroup [40]. It is recommended to have future trials to directly assess the cardiovascular effects of TNF inhibitors, especially in patients with heart failure.

IL-12/23 Inhibitors, IL-23 Inhibitors, and IL-17 Inhibitors

The cardiovascular effects of IL-12/23 inhibitors (e.g., ustekinumab and briakinumab) remain controversial. Some analyses have suggested a potential increase in MACE, particularly in patients with high baseline cardiovascular risk shortly after initiating treatment [12,41], whereas few studies show otherwise [39]. Ustekinumab has shown transient vascular anti-inflammatory effects, including short-term reductions in aortic vascular inflammation and inflammatory cytokines [12,39], though these effects may not be sustained long-term. Briakinumab reported safety concerns of an increased risk of MACE during the initial analyses, which led to trial discontinuation in 2011 [39]. Also, an increased rate of MACE in patients treated with anti-IL-23 therapy was confirmed in a meta-analysis of randomized clinical trials, but that mainly covered individuals with high cardiovascular risk [10].

In contrast, IL-17 inhibitors appear to offer more consistent cardiovascular benefits. Studies have demonstrated reductions in non-calcified plaque burden, lipid-rich necrotic core volume, and coronary inflammation, with the most notable improvements observed in patients treated with anti-IL-17 agents compared to other biologics or conventional therapies [39,41]. Recent trials reported improved endothelial, myocardial, and vascular function with IL-17 inhibition. However, not all trials found significant improvements in subclinical atherosclerosis, indicating variability in outcomes [41]. Hence, due to the short duration and limited event numbers in many studies, further long-term research is needed to clarify the cardiovascular safety profile of IL-17 inhibitors.

Methotrexate

Methotrexate has been associated with consistent cardiovascular risk reduction when used for inflammatory disorders, as evidenced by various studies, including one reporting a 21% reduction in overall CVD risk and an 18% lower risk of myocardial infarction in a comprehensive meta-analysis [38], along with other studies that showed non-significant trends toward cardiovascular benefit. However, limitations such as selection bias, reliance on clinician-reported outcomes, and lack of adjustment for disease severity highlight the need for more robust evidence [42].

Other Therapies

Janus kinase (JAK) inhibitors have demonstrated variable cardiovascular safety profiles depending on dose and indication. High-dose tofacitinib in rheumatoid arthritis patients with multiple cardiovascular risk factors has been linked to an increased risk of venous thromboembolism, prompting a black box warning by regulatory authorities [40]. However, a meta-analysis of dermatologic use of JAK and signal transducer and activator of transcription (JAK-STAT) inhibitors over a median duration of 16 weeks reported no significant increase in MACE or venous thromboembolism, and they were generally well tolerated [43]. In contrast, a large randomized safety trial in rheumatoid arthritis revealed higher incidences of MACE, cancer, and opportunistic infections with both low- and high-dose tofacitinib compared to TNF inhibitors, without meeting the noninferiority criteria for cardiovascular outcomes [38]. These findings highlight the need for indication- and dose-specific risk assessment when prescribing JAK inhibitors.

Corticosteroid use has been consistently associated with an elevated risk of cardiovascular events, including myocardial infarction, stroke, heart failure, and MACE, particularly at doses exceeding 7.5 mg/day of prednisone or equivalent [38]. While corticosteroids are effective in managing chronic inflammatory diseases, they exacerbate key atherosclerotic risk factors such as hypertension, glucose intolerance, dyslipidemia, and obesity, contributing to premature coronary artery disease [7]. High-dose or prolonged glucocorticoid therapy has also been linked to increased risks of atrial fibrillation, heart failure, and ischemic heart disease, with risk correlating to both dosage and timing of exposure [44]. Although findings on lipid effects are mixed, the overall cardiovascular burden underscores the need for cautious use and routine risk monitoring in patients receiving corticosteroids.

In managing CVD in patients with ADs, a comprehensive approach that includes ACE inhibitors, beta-blockers, and statins is critical not only for controlling blood pressure and lipid levels but also for modulating the underlying inflammatory and immune processes that drive both CVD and autoimmunity [45]. ACE inhibitors reduce angiotensin II-mediated vasoconstriction and inflammation, thereby protecting vascular endothelium, while beta-blockers mitigate catecholamine-induced cardiac stress and exert anti-inflammatory effects by dampening sympathetic overactivity. Statins, beyond their lipid-lowering properties, provide pleiotropic benefits by reducing systemic inflammation, improving endothelial function, and modulating immune cell activity, potentially shifting the immune response away from a pro-inflammatory phenotype. These pharmacologic interventions, when combined with disease-modifying antirheumatic drugs (DMARDs) or biologics targeting autoimmune activity, contribute to reducing long-term cardiovascular morbidity and mortality in this high-risk population [45,46].

Non-pharmacologic Interventions

Beyond biologics, targeted lifestyle and adjunctive therapies play a pivotal role in mitigating this risk. Caloric restriction in overweight psoriasis patients drives both CV [10] and skin improvement [41], along with a reduction in the Psoriasis Area and Severity Index (PASI) score [41,47]. A meta-analysis of seven randomized controlled trials (RCTs) (>900 patients) showed that weight loss tripled skin clearance rates when paired with standard therapies. The Mediterranean diet, endorsed by the National Psoriasis Foundation (NPF), reduces hs-CRP and fat mass and improves disease severity [40,41]. Smoking cessation improves both psoriasis severity and cardiovascular outcomes. Chronic stress worsens inflammation and CVD, creating a feedback loop. Interventions like yoga, exercise, and relaxation techniques offer low-cost, high-impact relief, particularly vital where access to advanced therapies is limited [41].

In a retrospective study of patients (median of 4.3 years), statin therapy was associated with a reduction in incident myocardial infarction. Psoriasis patients on high-intensity statins displayed a similar reduction in lipids and cardiovascular events [40]. Proprotein convertase subtilisin/kexin (PCSK)9 inhibitors add potential anti-inflammatory benefits and merit further exploration [41]. Traditionally, for diabetes, metformin improves psoriasis severity (PASI) and metabolic health via AMPK activation. Promising but underutilized, it lacks clinical validation and needs further evaluation [41].

A centralized care coordination model was proposed where dermatologists or rheumatologists screen psoriasis patients for CVD risk and refer them to an NPF care coordinator [48]. The coordinator calculates the 10-year atherosclerotic cardiovascular disease (ASCVD) risk and develops a tailored plan in collaboration with the primary care provider, as prior research has demonstrated that interventions utilizing care coordinators (CCs) for patients with comorbid diseases improve patient outcomes [48].

Given the cumulative cardiovascular burden of chronic psoriasis, adding up to a 1% absolute cardiovascular risk increase per year, dermatologists should proactively counsel patients on their elevated risk and urge timely engagement with primary care and cardiology for preventive management [40].

Sex differences in autoimmune CVD risk

Chronic systemic ADs are more common in women, with 61% of those affected being female [5]. Women are considered to be more susceptible to ADs compared to men because fluctuations in sex hormones during the menstrual cycle, menopause, or pregnancy can impact immune function [41]. 

Cardiovascular complications in females with rheumatologic conditions occur secondary to endothelial dysfunction, microvascular dysfunction, and osteosclerotic plaque formation.

Women with chronic systemic autoimmune conditions can significantly increase the risk of CVD. For example, of the risks and predictors for mortality in a cohort of 152 consecutive outpatients with rheumatoid arthritis, the standardized mortality ratio (SMR) was 1.61 for women, 1.52 for men, and 1.56 for both sexes combined. CVD comorbidity was a significant predictor of mortality in this study [49].

Females also experience a higher level of psychological and social stressors, which can significantly impact immune function and predispose them to more AD [7]. African American women with a history of depression were associated with more significant organ damage [7].

Women with the most severe organ damage have the greatest need for social support. Therefore, it's important to address the psychosocial stress and societal factors in women with rheumatological conditions [7]. Given the complex interaction between hormone changes and environmental factors, surveillance and management of a female patient with an autoimmune condition deserve more special attention [7].

Multidisciplinary care teams and more interventions with a focus on improving social support to females with chronic autoimmune dysfunction can lessen the extent of cardiovascular events [7]. Physicians need to identify and treat cardiovascular risk factors in women with autoimmune conditions early, and testing with echocardiography and coronary artery calcium score can be considered as appropriate [7]. Therefore, early identification of CVD risk factors in women with chronic autoimmune conditions can help reduce further complications [7].

Knowledge gaps and future directions

While our understanding is growing, several key knowledge gaps persist, underscoring the need for focused future directions.

Firstly, a deeper mechanistic understanding of disease-specific cardiovascular pathology is crucial. While general inflammatory pathways are recognized, the precise differential impact of specific ADs (e.g., autoantibodies in SLE vs. cytokine profiles in psoriasis) on diverse cardiovascular manifestations (e.g., accelerated atherosclerosis, direct myocardial injury, valvular disease) remains largely undefined. Future research should delve into how unique immune cell subsets and their mediators contribute to specific vascular injury patterns. Biomarker discovery, including epigenetic modifications and metabolomic profiles, is essential to predict distinct cardiovascular outcomes in different AD cohorts [20,25].

Secondly, refining CVD risk stratification tools for AD patients is a significant unmet need. Traditional risk calculators consistently underestimate risk, and the current blanket adjustment for ADs is insufficient. Development of disease-specific CVD risk prediction models is needed, incorporating inflammatory markers, disease activity scores, autoantibody profiles, and advanced imaging parameters like carotid intima-media thickness and coronary artery calcification [15]. Large-scale prospective studies are required to validate these novel tools and explore the integration of machine learning and artificial intelligence (AI) for more precise, personalized risk algorithms.

Thirdly, optimizing therapeutic strategies for dual disease management demands further investigation. While immunomodulatory therapies show promise in mitigating cardiovascular risk, definitive head-to-head comparative effectiveness studies on long-term cardiovascular outcomes are lacking. The full cardiovascular safety and benefit profiles of newer biologics and JAK inhibitors are still under investigation, with concerns regarding agents like IL-12/23 inhibitors and high-dose tofacitinib [37,50]. Future research should focus on personalized treatment approaches, considering individual inflammatory endotypes and baseline CVD risk. Evaluating integrated care models, fostering collaboration between specialists, and exploring adjunctive therapies (e.g., metformin, PCSK9 inhibitors) are also vital [21,41].

Finally, addressing health disparities and patient-centered outcomes is paramount. The review notes women's increased predisposition, but other disparities (e.g., race, ethnicity, socioeconomic status) that influence both AD severity and access to CVD care require more attention. Future research must investigate these disparities, identify barriers, and develop targeted interventions to promote health equity. Incorporating patient-reported outcomes (PROs) will provide a more holistic understanding of the impact of CVD and its management on AD patients' quality of life [41]. By addressing these gaps, future research can pave the way for more precise, personalized, and equitable management of cardiovascular risk in ADs.

## Conclusions

Patients with ADs face unique cardiovascular risks driven by chronic inflammation and autoimmunity. An integrated management approach that targets both immunologic control and traditional risk factors is essential to reduce this burden. The findings emphasize the importance of cardiovascular risk assessment and prevention as integral to AD care pathways. Continued research is needed to clarify causal pathways and to test whether emerging therapies targeting inflammatory mechanisms can prevent cardiovascular events. Future work should explore personalized risk stratification using genetic, biomarker, and imaging data to guide therapy. Collaborative care models between rheumatologists and cardiologists may help translate emerging evidence into improved patient outcomes. Large prospective studies and controlled trials will be important to validate these strategies and refine clinical guidelines.

## References

[1] Weber B, Merola JF, Husni ME, Di Carli M, Berger JS, Garshick MS (2021). Psoriasis and cardiovascular disease: novel mechanisms and evolving therapeutics. Curr Atheroscler Rep.

[2] Wilkinson MJ, Shapiro MD (2024). Immune-mediated inflammatory diseases, dyslipidemia, and cardiovascular risk: a complex interplay. Arterioscler Thromb Vasc Biol.

[3] Furman D, Campisi J, Verdin E (2019). Chronic inflammation in the etiology of disease across the life span. Nat Med.

[4] Barozet M, Le Tilly O, Bejan-Angoulvant T, Fesler P, Roubille C (2024). Hypertension and cardiovascular outcomes in inflammatory and autoimmune diseases: a systematic review and meta-analysis. Curr Hypertens Rep.

[5] Conrad N, Verbeke G, Molenberghs G (2022). Autoimmune diseases and cardiovascular risk: a population-based study on 19 autoimmune diseases and 12 cardiovascular diseases in 22 million individuals in the UK. Lancet.

[6] Huang WC, Hsieh SC, Wu YW (2023). 2023 Taiwan Society of Cardiology (TSOC) and Taiwan College of Rheumatology (TCR) joint consensus on connective tissue disease-associated pulmonary arterial hypertension. Acta Cardiol Sin.

[7] Mehta PK, Levit RD, Wood MJ (2023). Chronic rheumatologic disorders and cardiovascular disease risk in women. Am Heart J Plus.

[8] Berger M, Fesler P, Roubille C (2021). Arterial stiffness, the hidden face of cardiovascular risk in autoimmune and chronic inflammatory rheumatic diseases. Autoimmun Rev.

[9] Tokuyama M, Mabuchi T (2020). New treatment addressing the pathogenesis of psoriasis. Int J Mol Sci.

[10] Cai J, Cui L, Wang Y, Li Y, Zhang X, Shi Y (2021). Cardiometabolic comorbidities in patients with psoriasis: focusing on risk, biological therapy, and pathogenesis. Front Pharmacol.

[11] Xie F, Chen L, Yun H, Levitan EB, Curtis JR (2021). Benefits of methotrexate use on cardiovascular disease risk among rheumatoid arthritis patients initiating biologic disease-modifying antirheumatic drugs. J Rheumatol.

[12] Branisteanu DE, Nicolescu AC, Branisteanu DC (2022). Cardiovascular comorbidities in psoriasis (review). Exp Ther Med.

[13] Aviña-Zubieta JA, Choi HK, Sadatsafavi M, Etminan M, Esdaile JM, Lacaille D (2008). Risk of cardiovascular mortality in patients with rheumatoid arthritis: a meta-analysis of observational studies. Arthritis Rheum.

[14] Hansson GK (2005). Inflammation, atherosclerosis, and coronary artery disease. N Engl J Med.

[15] Agca R, Heslinga SC, Rollefstad S (2017). EULAR recommendations for cardiovascular disease risk management in patients with rheumatoid arthritis and other forms of inflammatory joint disorders: 2015/2016 update. Ann Rheum Dis.

[16] Mehta NN, Yu Y, Pinnelas R, Krishnamoorthy P, Shin DB, Troxel AB, Gelfand JM (2011). Attributable risk estimate of severe psoriasis on major cardiovascular events. Am J Med.

[17] Gelfand JM, Neimann AL, Shin DB, Wang X, Margolis DJ, Troxel AB (2006). Risk of myocardial infarction in patients with psoriasis. JAMA.

[18] Libby P, Ridker PM, Hansson GK (2009). Inflammation in atherosclerosis: from pathophysiology to practice. J Am Coll Cardiol.

[19] Mehta NN, Azfar RS, Shin DB, Neimann AL, Troxel AB, Gelfand JM (2010). Patients with severe psoriasis are at increased risk of cardiovascular mortality: cohort study using the General Practice Research Database. Eur Heart J.

[20] Porsch F, Binder CJ (2024). Autoimmune diseases and atherosclerotic cardiovascular disease. Nat Rev Cardiol.

[21] Prasad M, Hermann J, Gabriel SE (2015). Cardiorheumatology: cardiac involvement in systemic rheumatic disease. Nat Rev Cardiol.

[22] Han C, Robinson DW, Hackett MV, Paramore LC, Fraeman KH, Bala MV (2006). The prevalence ratio of ischemic heart disease. J Rheumatol.

[23] Urowitz MB, Gladman DD (2000). Accelerated atheroma in lupus-background. Lupus.

[24] Harrington CL, Dey AK, Yunus R, Joshi AA, Mehta NN (2017). Psoriasis as a human model of disease to study inflammatory atherogenesis. Am J Physiol Heart Circ Physiol.

[25] López-Pedrera C, Pérez-Sánchez C, Ramos-Casals M, Santos-Gonzalez M, Rodriguez-Ariza A, Cuadrado MJ (2012). Cardiovascular risk in systemic autoimmune diseases: epigenetic mechanisms of immune regulatory functions. Clin Dev Immunol.

[26] Chen IW, Wang WT, Lai YC, Lin CM, Liu PH, Wu SZ, Hung KC (2024). Association between systemic sclerosis and risk of cerebrovascular and cardiovascular disease: a meta-analysis. Sci Rep.

[27] Chaturvedi S, Braunstein EM, Yuan X (2020). Complement activity and complement regulatory gene mutations are associated with thrombosis in APS and CAPS. Blood.

[28] Victor VM, Rocha M, Solá E, Bañuls C, Garcia-Malpartida K, Hernández-Mijares A (2009). Oxidative stress, endothelial dysfunction and atherosclerosis. Curr Pharm Des.

[29] Han C, Robinson DW Jr, Hackett MV, Paramore LC, Fraeman KH, Bala MV (2006). Cardiovascular disease and risk factors in patients with rheumatoid arthritis, psoriatic arthritis, and ankylosing spondylitis. J Rheumatol.

[30] Durante A, Bronzato S (2015). The increased cardiovascular risk in patients affected by autoimmune diseases: review of the various manifestations. J Clin Med Res.

[31] Huang WC, Hsu CH, Sung SH (2019). 2018 TSOC guideline focused update on diagnosis and treatment of pulmonary arterial hypertension. J Formos Med Assoc.

[32] Anyfanti P, Dara A, Angeloudi E, Bekiari E, Dimitroulas T, Kitas GD (2021). Monitoring and managing cardiovascular risk in immune mediated inflammatory diseases. J Inflamm Res.

[33] Abohelwa M, Kopel J, Shurmur S, Ansari MM, Awasthi Y, Awasthi S (2023). The Framingham study on cardiovascular disease risk and stress-defenses: a historical review. J Vasc Dis.

[34] Curtis JR, Xie F, Crowson CS (2020). Derivation and internal validation of a multi-biomarker-based cardiovascular disease risk prediction score for rheumatoid arthritis patients. Arthritis Res Ther.

[35] Drosos GC, Vedder D, Houben E (2022). EULAR recommendations for cardiovascular risk management in rheumatic and musculoskeletal diseases, including systemic lupus erythematosus and antiphospholipid syndrome. Ann Rheum Dis.

[36] Esdaile JM, Abrahamowicz M, Grodzicky T (2001). Traditional Framingham risk factors fail to fully account for accelerated atherosclerosis in systemic lupus erythematosus. Arthritis Rheum.

[37] Galajda NÁ, Meznerics FA, Mátrai P (2024). Reducing cardiovascular risk in immune-mediated inflammatory diseases: tumour necrosis factor inhibitors compared to conventional therapies-a systematic review and meta-analysis. J Eur Acad Dermatol Venereol.

[38] Roubille C, Richer V, Starnino T (2015). The effects of tumour necrosis factor inhibitors, methotrexate, non-steroidal anti-inflammatory drugs and corticosteroids on cardiovascular events in rheumatoid arthritis, psoriasis and psoriatic arthritis: a systematic review and meta-analysis. Ann Rheum Dis.

[39] Jiang Y, Chen Y, Yu Q, Shi Y (2023). Biologic and small-molecule therapies for moderate-to-severe psoriasis: focus on psoriasis comorbidities. BioDrugs.

[40] Garshick MS, Ward NL, Krueger JG, Berger JS (2021). Cardiovascular risk in patients with psoriasis: JACC review topic of the week. J Am Coll Cardiol.

[41] Mehta H, Narang T, Dogra S, Handa S, Hatwal J, Batta A (2024). Cardiovascular considerations and implications for treatment in psoriasis: an updated review. Vasc Health Risk Manag.

[42] Westlake SL, Colebatch AN, Baird J (2010). The effect of methotrexate on cardiovascular disease in patients with rheumatoid arthritis: a systematic literature review. Rheumatology (Oxford).

[43] Ireland PA, Jansson N, Spencer SK, Braden J, Sebaratnam D (2024). Short-term cardiovascular complications in dermatology patients receiving JAK-STAT inhibitors: a meta-analysis of randomized clinical trials. JAMA Dermatol.

[44] Liu D, Ahmet A, Ward L (2013). A practical guide to the monitoring and management of the complications of systemic corticosteroid therapy. Allergy Asthma Clin Immunol.

[45] Amaya-Amaya J, Montoya-Sánchez L, Rojas-Villarraga A (2014). Cardiovascular involvement in autoimmune diseases. Biomed Res Int.

[46] Syngle A, Verma I, Krishan P, Syngle V (2016). Disease-modifying antirheumatic drugs improve cardiovascular autonomic neuropathy in psoriatic arthritis. Ther Adv Musculoskelet Dis.

[47] Gisondi P, Bellinato F, Girolomoni G, Albanesi C (2020). Pathogenesis of chronic plaque psoriasis and its intersection with cardio-metabolic comorbidities. Front Pharmacol.

[48] Song WB, Garshick MS, Barbieri JS (2024). A care coordination model to prevent cardiovascular events in patients with psoriatic disease: a multicenter pilot study. J Invest Dermatol.

[49] Han K, Kim JH (2015). Transarterial chemoembolization in hepatocellular carcinoma treatment: Barcelona Clinic Liver Cancer staging system. World J Gastroenterol.

[50] Ytterberg SR, Bhatt DL, Mikuls TR (2022). Cardiovascular and cancer risk with tofacitinib in rheumatoid arthritis. N Engl J Med.

